# Iris Microcirculation After Selective Laser Trabeculoplasty: A Pilot Optical Coherence Tomography Angiography Study

**DOI:** 10.3390/vision9010021

**Published:** 2025-03-05

**Authors:** Dmitrii S. Maltsev, Alexey N. Kulikov, Alina A. Kazak

**Affiliations:** Department of Ophthalmology, Military Medical Academy, St. Petersburg 194044, Russia; alexey.kulikov@mail.ru (A.N.K.); alinakazak96@gmail.com (A.A.K.)

**Keywords:** open-angle glaucoma, intraocular pressure, optical coherence tomography angiography, selective laser trabeculoplasty

## Abstract

Background: This research was conducted to study changes in iris microcirculation using optical coherence tomography angiography (OCTA) in patients with primary open-angle glaucoma after selective laser trabeculoplasty (SLT). Methods: All patients received standard SLT. OCTA examination of the iris was performed before SLT and one day and seven days after SLT using RTVue-XR with a 3 mm scan pattern and follow-up function. Iris vascularity was calculated with ImageJ software (version 1.53k) as vessel density on binarized images. Correlation between absolute or percentage changes in iris vessel density and intraocular pressure (IOP) changes was calculated. Results: A total of 31 eyes (31 patients, 10 females, 70.7 ± 8.9 years) were included. Iris vessel density increased statistically significantly (*p* = 0.002) the day after SLT followed by a decrease to baseline level at one week. A statistically significant correlation (r = 0.57, *p* = 0.002) was found between the percentage change in iris vessel density the day after the procedure and IOP change at three months. Conclusion: SLT is associated with a transitory increase in iris vessel density, which can be observed with OCTA the day after the procedure. Substantial increase in iris vascularity is associated with a poorer IOP-lowering effect of SLT in eyes with open-angle glaucoma.

## 1. Introduction

Selective laser trabeculoplasty (SLT), first introduced in 1995 by Latina and Park, has become one of the most popular options for laser treatment of open-angle glaucoma. SLT can be used as both an individual method to control intraocular pressure (IOP) or in combination with topical antiglaucoma medications. SLT is based on selective photothermolysis of melanin in the trabecular meshwork by delivering short pulses of a 532 nm Nd:YAG laser. SLT causes no thermal damage to nonpigmented cells and is considered as a tissue sparing procedure, which can be repeated as needed. This procedure can reduce IOP by up to 30.0% from baseline, with optimal results considered as around 20.0%. This reduction in IOP may last for years after the procedure; however, a suboptimal or no IOP-lowering effect is observed in up to 25.0% of eyes [1]. The IOP-lowering effect of SLT was shown to be unrelated to age, sex, hypertension, family history of glaucoma, previous anterior segment surgery, laser energy used, type of open-angle glaucoma, degree of trabecular meshwork pigmentation, or local antiglaucoma therapy [2,3]. At the same time, according to several studies, baseline IOP was highly significant for the decrease in IOP after SLT [3,4]. Additionally, anterior chamber particles, representing cellular debris and pigmentary granules, and Schlemm’s channel remodeling, both observed early after SLT, were predictive for favorable response [5,6].

A procedure in the anterior chamber SLT can potentiate an inflammatory response. This could be explained by the increase in cytokine expression after SLT [7] and some similarity in the effects of SLT and prostaglandin analogs on the permeability of cultured Schlemm’s channel cells [8]. Although some symptoms and findings after SLT, including ocular discomfort, photophobia, redness, and cells in the anterior chamber [9], agree with these observations, cases of clinically significant anterior segment inflammation are quite rare [10]. Anti-inflammatory medications, including NSAIDs, do not affect the IOP-lowering effects of SLT. All of this suggests rather a minor role for inflammation in the mechanisms of action of SLT; however, this does not exclude subclinical inflammation after SLT. At the same time, clinical studies of inflammation after SLT are however limited by biomicroscopical examination and laser flare photometry, although the latter has demonstrated that SLT does not induce inflammation in the anterior chamber [11].

Recent advances in optical coherence tomography angiography (OCTA) have enabled its application to the anterior segment of the eye where it can be used to assess microcirculation of the iris, limbus, or conjunctiva. OCTA has shown that the iris vasculature is closely correlated with the anterior chamber cells where inflammatory activity is revealed as high vessel reflectance on the image [12]. We believe that OCTA may shed light on the changes in iris microcirculation after SLT, which may serve as a biomarker of anterior segment inflammation. The aim of this study was to assess the changes in iris microcirculation after SLT using OCTA.

## 2. Materials and Methods

In this prospective cohort study, we included patients with mild and moderate primary open-angle glaucoma receiving more than one antiglaucoma medication who had not achieved target IOP. The assessment of the severity of the glaucoma was based on characteristic visual field loss and optic nerve damage. The exclusion criteria were narrow anterior chamber angle, decreased corneal transparency, baseline IOP more than 30.0 mmHg, ocular or systemic vascular diseases, and advanced glaucoma. The limit on the maximum IOP included was based on the potential effect of high IOP on iris perfusion, influencing changes caused by SLT. Eyes with highly pigmented irises affecting OCTA imaging or cases with poor scan quality were also excluded. Patients were prohibited from the use of NSAIDs over a month after SLT.

The study followed the ethical standards laid down in the Declaration of Helsinki and was approved by the Local Ethics Committee (extract from protocol #232 from 18 February 2020). All patients received standard ophthalmic examination including Goldmann applanation tonometry and OCTA examination of the iris at baseline. OCTA examination was performed with RTVue-XR (Optovue, Fremont, CA, USA) using 3 mm Angio Retina scan manually setting focus and z-position of the scanning beam at the maximum values. All examinations were performed in standard photopic conditions to compensate for differences in pupil size using the follow-up function.

All SLT procedures were performed by a single experienced operator (KAA) under the topical anesthesia with one drop of oxybuprocaine 0.4% (Sentiss, Uster, Switzerland) using an SLT laser system Tango (Ellex, Adelaide, SA, Australia). SLT was performed in the standard manner and included approximately 100 shots applied within 360 degrees of the anterior chamber with a shot energy of 0.6 to 0.8 mJ. No IOP-lowering medication was changed during the follow-up period. No NSAIDs were used in this study. IOP measurements and biomicroscopy were performed one day, one week, one month, and three months after the procedure. Iris microcirculation was assessed one day and one week after the procedure. Long-term changes were not analyzed as iris microcirculation returned to the normal range within one week after SLT.

To analyze iris microvasculature, the full thickness slab between the anterior and posterior iris surface was chosen. Segmentation lines were inspected to capture the entire iris thickness and were corrected if needed. If the pupil diameter between two visits differed by more than 10% (measured on cross sectional scan crossing the pupil center), the case was excluded from analysis.

**Figure 1:**
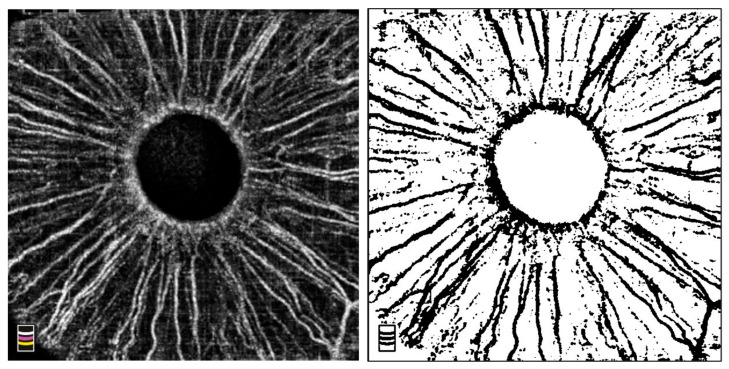
Representative example of iris microcirculation displayed with optical coherence tomography angiography before (**left**) and after (**right**) binarization. Vessel density = 25.5%.

Next, OCTA images were exported and uploaded in ImageJ software (version 1.53k) (NIH, Bethesda, MD, USA). All images were converted to 8-bit format, followed by the application of a default auto-thresholding algorithm. After thresholding, the images were binarized. The “Analyze particles” function was used to calculate the total flow signal area for the entire iris area, which was converted to the vessel density calculated as a ratio of total flow area to the scan area (Figure 1).

Statistical analysis was performed with MedCalc 18.4.1 (MedCalc Software, Ostend, Belgium). The results are presented as the mean ± standard deviation. Spearman correlation coefficient was used to evaluate the association between IOP, or its changes, and iris vessel density at different time points. Repeated measures analysis of variance was used to compare iris vessel density before and after SLT. ROC analysis was performed to assess the performance of percentage change in iris vessel density to discriminate eyes with an IOP-lowering effect of −10.0% from baseline or less. Statistical significance was defined as *p* < 0.05.

## 3. Results

A total of 31 eyes (31 patients, 10 females and 21 males, 70.7 ± 8.9 years) with open-angle glaucoma were included. There were 18 patients with mild and 13 patients with moderate open-angle glaucoma. Twenty patients received a fixed-dose combination of prostaglandin analogs and beta-blockers, nine patients received a fixed-dose combination of carbonic anhydrase inhibitor and beta-blockers, and two patients received prostaglandin analogs and an alpha-2 adrenergic receptor agonist. All eyes demonstrated mild to moderate pigmentation of trabecular meshwork. The mean baseline and final IOP was 21.1 ± 3.9 and 16.8 ± 3.8 mmHg (*p* < 0.001). The mean decrease in IOP was −4.2 ± 3.3 mmHg. No correlation was found among the baseline parameters, including baseline IOP, age, severity of glaucoma or topical medications, and baseline iris vessel density (*p* > 0.05). In the multiple regression model, age, glaucoma severity, and baseline IOP showed no statistically significant association with baseline iris vessel density (*p* > 0.05).

Vessel density of the iris at baseline, one day, and one week after SLT was 26.9 ± 6.5%, 31.6 ± 7.9%, and 27.4 ± 7.9%, respectively. One day after SLT, iris vessel density had increased statistically significantly (*p* = 0.002), followed by a decrease to baseline level at one week after SLT. No statistically significant difference was found between baseline and one week iris vessel density (*p* = 0.25). No changes in the vascularity of the iris were detected with slit-lamp examination.

There was a negative correlation between the baseline IOP and decrease in IOP (r = −0.79, *p* < 0.001) but no correlation was found between the baseline and IOP at three months (r = 0.25, *p* = 0.22).

No statistically significant correlation was found between IOP at three months and the parameters of iris vessel density, including baseline iris vessel density, iris vessel density the day after the procedure, or change in iris vessel density. Three months after SLT, a statistically significant correlation (Table 1) was found between the change in iris vessel density and change in IOP.

**Figure 2:**
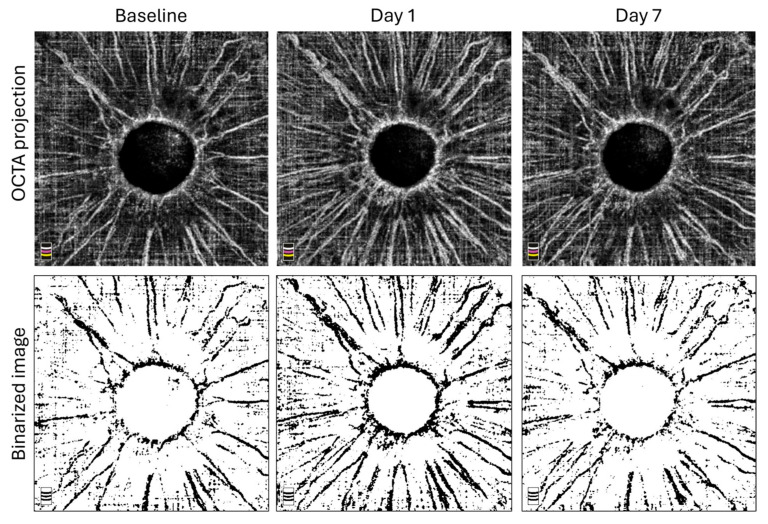
Optical coherence tomography angiography of iris in patient with minimal IOP-lowering effect after selective laser trabeculoplasty. Baseline, day 1, and day 7 iris vessel density was 24.1%, 30.7%, and 24.9%, respectively. IOP-lowering effect at three months was −0.3 mmHg.

The highest correlation was found between percentage of IOP reduction and percentage changes in iris vessel density (r = 0.57, *p* = 0.002) (Figure 2). ROC analysis of percentage of iris vessel density change in predicting IOP lowering effect less than −10.0% showed AUC = 0.94 (sensitivity = 87.5%; specificity = 88.9%)

## 4. Discussion

In this study, we showed that SLT in eyes with open-angle glaucoma is followed by a temporary increase in iris vascularity, which can be identified using OCTA the day after the procedure and which almost completely regressed to baseline level at one week after SLT. This change in the vascularity of the iris was not detectable with slit-lamp examination and represents a subclinical phenomenon. The magnitude of the increase in iris vessel density was negatively associated with long-term IOP-lowering effects of SLT. In cases of substantial increase in iris vessel density, the magnitude that IOP decreases at three months after SLT was low.

OCTA is a relatively new diagnostic tool in ophthalmology that allows for the noninvasive imaging of the retinal microcirculation with high resolution. Since some OCTA devices have a wide focusing range, the technology can be adapted to visualize the anterior eye segment [13]. The use of OCTA to display the microcirculation of the pterygia, pinguecula, limbus, conjunctiva, and iris has been described in a number of studies [14,15,16,17]. For the iris, OCTA may be used to evaluate anterior segment inflammation. Although the repeatability of OCTA imaging of the iris is relatively high, at least two factors significantly affect OCTA imaging of the iris: iris pigmentation and pupil diameter. Since melanin effectively blocks the OCT scanning beam, brown eyes show only a limited number of vessels. Therefore, the magnitude of changes in iris vascularity in these eyes is naturally limited. To avoid this effect, in our study, we included only patients with blue or gray irises. To compensate for the variability of pupil diameter, we performed all examinations in the standard photopic condition with the same artificial light source.

Based on the changes observed, we suggest several potential reasons for the transitory increase in iris vessel density. Firstly, the increase may be caused by collateral iris damage by the laser beam. Indeed, the laser beam runs tangentially to the iris surface and can touch it during pulses. However, cases with a shallow anterior chamber were not included in the study. Moreover, during manipulation, the operator conventionally controls the profile of the laser spot to keep it round and avoid interaction of the laser beam with the iris surface. Therefore, we exclude the role of direct iris damage. Secondly, hyperemia of the iris may be induced indirectly by the activation of biological signaling pathways and the expression of biologically active molecules produced by the trabecular meshwork [18,19]. However, most of the biological effects of SLT were shown in vitro and their actual role in SLT is still not fully understood. Thirdly, inflammation may be part of cyclitis caused by damage to the ciliary body, which seems possible due to the large spot used in SLT, which potentially covers a part of the ciliary body. However, except for a single study that showed shallow ciliochoroidal detachment after STL using ultrasound biomicroscopy, there are no data supporting changes in the ciliary body after SLT [20]. Certain types of anterior uveitis (such as those related to HLA B-27) may present with acute hypotony possibly due to the increase in uveoscleral outflow or aqueous production decrease [21,22]. Although anterior segment inflammation may cause a transient decrease in IOP, it would not influence long-term outcomes of SLT since inflammation and transitory IOP reduction regress a few days after SLT. The latter point can be observed in patients with a notable increase in iris vascularity. The stable IOP decrease in such cases results from SLT facilitating the outflow thorough the trabecular meshwork, not from changes in the production of aqueous humor. Otherwise, damage to the ciliary body may be possible in some eyes due to anatomical predisposition in terms of structure of the anterior chamber angle. In general, the results of our study suggest that, although inflammation of anterior eye segment seems to be present in eyes after SLT, it plays no, or only a minor role, as the mechanism of action of SLT. This corresponds to the absence of any effect of topical NSAIDs on the IOP-lowering effect of SLT. Nevertheless, independent of the exact cause underlying changes in iris perfusion, vessel dilation is the only mechanism which may immediately increase perfusion. This may occur through the dilation of apparently visible vessels which become wider and therefore occupy a larger area, and through the appearance of new vessels where flow signal was previously poorly detectable due to slow blood flow. The sensitivity of OCTA to blood flow velocity supports the second mechanism. The concept of vessel dilation as a direct mechanism of increase in iris perfusion agrees with similar changes registered with OCTA in anterior uveitis [1].

Alternatively, we suggest that a decrease in IOP may facilitate blood flow and modify iris perfusion that may result in an increase in iris vessel density. However, we found no correlation between baseline IOP and baseline iris vessel density, or between change in IOP at the day after SLT and increase in iris vessel density. In the context of the relationship between IOP and iris perfusion, ocular perfusion pressure could be informative but was not assessed in this study. We did not analyze long-term changes in iris microcirculation since it returned to baseline range within one week after SLT. Moreover, the aim of this study was to assess the iris microcirculation changes directly associated with SLT, while long-term changes can be associated with IOP changes.

Since patients with a greater increase in iris vessel density show poorer long-term-outcomes, the next-day increase in iris vascularity may be used as a biomarker for SLT prognostication. Although the duration of this study is limited to three months, it seems unlikely that patients with the next-day increase in iris vascularity and poor IOP-lowering effect at three months will show a decline of IOP later than three months. Therefore, the assessment of iris vascularity after SLT may be used to predict which patients will have an unsatisfactory outcome of SLT. Although baseline iris vascularity may potentially depend on various factors, including age, severity of glaucoma, IOP, and medication we did not find any association with these factors. This is to be expected since OCTA imaging of iris microcirculation is highly sensitive to pigmentation degree and, therefore, even after excluding dark eyes, we still cannot exclude the effects of iris pigmentation on the vascularity metric.

This pilot study has several limitations. Firstly, it included a relatively small number of study participants. Secondly, although we suggest a role for the ciliary body and cyclitis in the transitory increase in iris vascularity, this conjecture requires further studies based on ultrasound biomicroscopy. Thirdly, heavily pigmented brown eyes were excluded from this study due to the inability of OCTA to sufficiently display iris microcirculation in such eyes. Therefore, we cannot extrapolate our results to the entire population. Fourthly, we did not use any control methods, such as laser flare meter, which would assess the actual level of anterior chamber inflammation in relation to the changes in iris vascularity. Finally, the patients were receiving antiglaucoma medication, which may have affected iris microcirculation or changes in iris vessel density in response to SLT. Although we did not find any association between medications prescribed and changes in iris vessel density after SLT, further studies in treatment naïve eyes are required. Due to potential effects on iris microcirculation, we did not use pilocarpine before SLT and OCTA examination, albeit this would allow us to minimize differences in pupils size.

## 5. Conclusions

In conclusion, this study showed that SLT is accompanied by transitory increase in iris vessel density, which may be registered by OCTA and used for prognostication of unsatisfactory outcomes. This transitory iris reaction is possibly related to short-term inflammation in the anterior segment, such as subclinical iritis or cyclitis; however, further studies are required for better understanding this phenomenon.

## Figures and Tables

**Table 1:** Relationships between intraocular pressure and iris vascularity.

	Baseline IOP	IOP Change	IOP Change %	Final IOP
Baseline iris vascularity	r = 0.22 *p* = 0.32	r = −0.22 *p* = 0.29	r = −0.21 *p* = 0.30	r = 0.03 *p* = 0.89
First day iris vascularity	r = −0.03 *p* = 0.82	r = 0.05 *p* = 0.68	r = 0.08 *p* = 0.55	r = 0.1 *p* = 0.63
Iris vascularity change	r = −0.38 *p* = 0.07	r = 0.44 *p* = 0.02	r = 0.49 *p* = 0.01	r = 0.16 *p* = 0.46
Iris vascularity change %	r = −0.44 *p* = 0.03	r = 0.52 *p* = 0.006	r = 0.57 *p* = 0.002	r = 0.17 *p* = 0.41

## Data Availability

The data presented in this study are available on request from the corresponding author. The data are not publicly available due to the not anonymized character of the dataset.

## Abbreviations

The following abbreviations are used in this manuscript:

OCTA	Optical coherence tomography angiography
SLT	Selective laser trabeculoplasty
NSAID	Non-steroidal anti-inflammatory drug
IOP	Intraocular pressure
